# Evolutionary dynamics of giant viruses and their virophages

**DOI:** 10.1002/ece3.600

**Published:** 2013-06-04

**Authors:** Dominik Wodarz

**Affiliations:** Department of Ecology and Evolutionary Biology, University of California321 Steinhaus Hall, Irvine, California, 92697

**Keywords:** Evolutionary dynamics, giant viruses, mathematical models, virophages

## Abstract

Giant viruses contain large genomes, encode many proteins atypical for viruses, replicate in large viral factories, and tend to infect protists. The giant virus replication factories can in turn be infected by so called virophages, which are smaller viruses that negatively impact giant virus replication. An example is Mimiviruses that infect the protist *Acanthamoeba* and that are themselves infected by the virophage Sputnik. This study examines the evolutionary dynamics of this system, using mathematical models. While the models suggest that the virophage population will evolve to increasing degrees of giant virus inhibition, it further suggests that this renders the virophage population prone to extinction due to dynamic instabilities over wide parameter ranges. Implications and conditions required to avoid extinction are discussed. Another interesting result is that virophage presence can fundamentally alter the evolutionary course of the giant virus. While the giant virus is predicted to evolve toward increasing its basic reproductive ratio in the absence of the virophage, the opposite is true in its presence. Therefore, virophages can not only benefit the host population directly by inhibiting the giant viruses but also indirectly by causing giant viruses to evolve toward weaker phenotypes. Experimental tests for this model are suggested.

## Introduction

Mimivirus (microbe mimicking virus) was first discovered in the water of a cooling tower in the United Kingdom, infecting the protist *Acanthamoeba*, and was shown to have characteristics that are atypical for the majority of viruses (La Scola et al. 2003; Raoult et al. 2004; Koonin 2005; Claverie et al. 2006; Suzan-Monti et al. 2006; Raoult and Forterre 2008; Claverie and Abergel 2009, 2010; Colson and Raoult 2010; Forterre 2010; Yamada 2011). It was found to be a dsDNA virus and was the largest virus known at the time. Its 1.2 Mb genome sequence contained more than 900 proteins with functions that are not normally associated with viruses, such as encoding crucial components of the protein translation machinery (Raoult et al. 2004). Unlike other viruses, it was visible with a light microscope (Claverie and Abergel 2010; Sun et al. 2010). Mimivirus is thought to be phylogenetically close to other large DNA viruses (Claverie and Abergel 2009). They replicate in large viral factories that are reminiscent of simple cell nuclei, resulting in the lysis of their *Acanthamoeba* host. A different strain of Mimivirus with a slightly larger genome, called Mamavirus, was found in a different cooling water in France (La Scola et al. 2008). In this case, an interesting discovery was the association of Mamavirus with a small satellite virus that was named Sputnik (La Scola et al. 2008). Sputnik replicates within the viral factories of Mimiviruses, using Mimivirus resources and consequently impairing Mimivirus replication, leading to the generation of defective Mimivirus particles (La Scola et al. 2008; Pearson 2008; Claverie and Abergel 2009; Desnues and Raoult 2010; Ruiz-Saenz and Rodas 2010; Sun et al. 2010; Desnues et al. 2012; Zhang et al. 2012). This also reduces Mimivius-induced lysis of amoebae. Therefore, Sputnik is a true “parasite” of Mimivirus rather than a regular satellite virus and has consequently been termed a “virophage,” although this distinction has been debated (Herrero-Uribe 2011; Krupovic and Cvirkaite-Krupovic 2011; Desnues and Raoult 2012; Fischer 2012).

Giant viruses and virophages are thought to be abundant in aquatic environments, infecting a variety of protists (Claverie et al. 2009; La Scola et al. 2010; Culley 2011; Yau et al. 2011). Consequently, virophages could play important roles in regulating the population dynamics between protists and their viruses. This has been examined in Antarctic lakes, where a relative of the Sputnik virophage was found to infect phycodnaviruses, which in turn infect phototrophic algae (Yau et al. 2011). In this system, data analysis and population models suggested that virophages reduce the mortality of algal cells and that they could have an important influence on the stability of microbial food webs.

The impact of virophages on the dynamics between giant viruses and their host cells is related to the effects of hyperparasites on parasite–host dynamics. Hyperparasites are defined as parasites that infect another parasite, leading to a food chain of parasitism. The effect of hyperparasitism on population dynamics has been examined in some detail with mathematical models (Beddington and Hammond 1977; May and Hassell 1981; Hochberg et al. 1990; Holt and Hochberg 1998), and the analysis often examined the impact on the biological control of insect pests. For example, Beddington and Hammond (1977) analyzed a scenario where a herbivore was infected by a parasite that was itself subject to infection by a hyperparasite. A recurrent result is that the introduction of a hyperparasite can reduce the effectiveness of biological control (Beddington and Hammond 1977; May and Hassell 1981). Because the primary parasite is attacked by the hyperparasite, the host/pest population benefits and can achieve higher equilibrium levels (Beddington and Hammond 1977; May and Hassell 1981). In addition, hyperparasites can influence the stability of a parasite–host system (Beddington and Hammond 1977). A detailed analysis of the stability of the food chain dynamics has been provided by Holt and Hochberg (1998), demonstrating both stabilizing and destabilizing effects. Related food web systems have been studied, including interactions among hosts, parasites, and predators, for example, Roy and Holt (2008).

Here, I build on these concepts and analyze mathematical models that describe the dynamics between a host protist, a virus infecting the protist, and a virophage infecting the virus. While the virophage is also a virus, for simplicity the term virus will be used to refer to the primary virus of the protist host, in order to distinguish it from the virophage. The model will be constructed with the *Acanthamoeba*–Mimivirus–Sputnik system in mind, although the model is quite general and also applicable to other systems. No population dynamic data exist so far to tailor the model to a specific system or to parameterize it. Instead, the general properties of the dynamics are investigated, in particular concentrating on the evolutionary dynamics of both the virus and the virophage. I will examine the evolution of “virophage pathogenicity,” that is, the degree to which the virophage inhibits replication of the primary virus. The model suggests that while selection favors a higher virophage pathogenicity, the emergence of more pathogenic virophages can also significantly destabilize the dynamics, rendering the system prone to extinction. Furthermore, the evolution of the primary virus population is investigated. It is found that the evolutionary trajectory of the primary virus can be changed by the presence of the virophage. While in isolation, the primary virus is expected to evolve toward higher basic reproductive ratios, the presence of the virophage can lead to the evolution of the primary virus to a reduced basic reproductive ratio. Experiments with the *Acanthamoeba*–Mimivirus–Sputnik system are suggested to test and refine the model, as well as to estimate para-meters.

## The Mathematical Models

We consider an ordinary differential equation model (ODE) that describes the average development of populations over time. These include the host *Acanthamoeba* population, *x*, amoebae infected with the Mimivirus, *y*_1_, and amoebae infected with the Mimivirus which in turn is infected with the Sputnik virophage, *y*_12_. Free virus is not explicitly taken into account. As the life span of viruses tends to be significantly shorter than that of cells, the virus populations are assumed to be in quasi steady state. The model is given by the following set of equations.


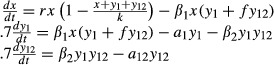
(1)

The amoeba population is characterized by logistic, density-dependent growth, described by the term *rx*(1*−*(*x+y*_1_*+y*_12_)/*k*). The intrinsic growth rate is given by *r* and the total amoeba population (uninfected + infected individuals) cannot exceed the carrying capacity *k*. Contact between the primary virus and uninfected amoeba cells leads to infection with a rate *β*_1_. The primary virus can be released from two sources. Obviously, one source is cells infected with the primary virus alone, *y*_1_. An additional source is cells that contain both the primary virus and the virophage, although they are likely to release the primary virus at a reduced rate. This is expressed by the parameter *f*, which describes the degree of primary virus inhibition by the virophage (i.e., the virophage “pathogenicity”) and can vary between zero and one. If *f* = 0, the primary virus cannot replicate at all in the presence of the virophage. If *f* = 1, the replication of the primary virus is not inhibited by the virophage. Amoeba infected with the primary virus only, *y*_1_, die with a rate *a*_1_ and become infected with virophage upon contact with a virophage-containing cell with a rate *β*_2_. Amoebae infected with both the primary virus and the virophage, *y*_12_, die with a rate *a*_12_. This death rate is determined both by the virophage and the primary virus. We assume that the primary virus contributes less to cell death in the presence compared with the absence of the virophage, due to inhibition of viral replication (parameter *f)*. In addition, the virophage itself can cause cell death with a rate *a*_ph_. Thus, the overall death rate of this cell population is given by *a*_12_ = *a*_ph_ + *fa*_1_. Note that while the reduced replication rate of the primary virus in virophage-infected cells is likely to be reflected in a reduced death rate of these cells, the death rate does not have to be decreased by the same factor, *f*, as written here. However, if the death rate of the infected cell is decreased by a different amount, expressed by an additional factor *g*, the results presented here do not change qualitatively. We do not track amoeba cells that are infected with the virophage only, as the virophage cannot replicate without the primary virus.

In order to address questions concerned with population extinction, we also consider a stochastic version of this model by applying the Gillespie algorithm to these ODEs (Gillespie 1977).

## Basic properties

The host amoeba population grows if *r* > 0 and reaches carrying capacity *k* in the absence of infection. The primary virus grows if its basic reproductive ratio is greater than one. This is given by 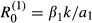. In this case, the system converges to the following equilibrium in the absence of the virophage:


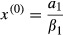



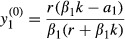



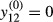


Note that the faster the replication rate of the primary virus is, *β*_1_, the lower the equilibrium number of infected cells. When a virophage is added to the system, it can establish an infection if its basic reproductive ratio is greater than one. It is given by 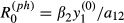. It is determined by the replication rate of the virophage and the death rate of infected cells, and also by the equilibrium number of cells infected by the primary virus in the absence of the virophage. As mentioned above, this is inversely proportional to the replication rate of the primary virus. Therefore, if the replication rate of the primary virus lies above a threshold, then 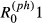 and the virophage fail to establish an infection. If the virophage does establish an infection, then the system converges to an equilibrium that is given by a very lengthy second-degree polynomial and hence not written out here.

**Figure 1:**
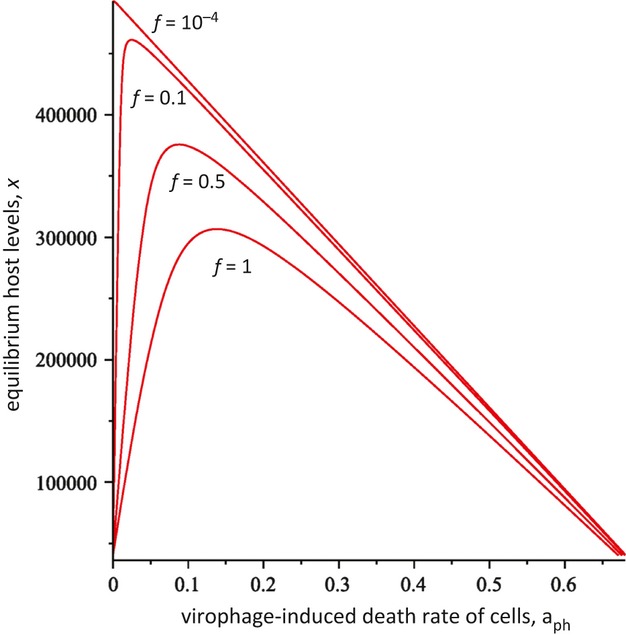
Effect of virophage-induced cell death, *a*_ph_, on the equilibrium host population, according to model (1). Different curves are shown, varying the virophage pathogenicity, *f*. Explanations are given in the text. Parameters were chosen as follows. *r* = 0.01; *β*_1_ = 2.5 × 10^−7^; *a*_1_ = 0.01; *β*_2_ = 2 × 10^−5^; *k* = 5 × 10^5^.

The dependence of the equilibrium population levels on the model parameters is largely intuitive. The host amoeba population is regulated by the primary virus, and the primary virus population is regulated by the virophage. Thus, a more effective virophage can downregulate the primary virus population, and this can in turn increase the equilibrium levels of the host amoebae, as described in previous studies on hyperparasitism (Beddington and Hammond 1977; May and Hassell 1981). However, because virophage-infected cells can also transmit the primary virus to host amoeba, virophage infection kinetics can at the same time lead to a reduction in the amoeba population, giving rise to a trade-off (Fig. 1). This is seen in the dependence of the equilibrium amoeba host population size on the death rate of virophage-infected cells. The lower the death rate of the cells, the larger the amount of virus released from these cells during their life span. The amount of successful primary virus replication in virophage-infected cells is determined by the parameter *f*. If the value of *f* is very low and close to zero, then primary virus replication is negligible in virophage-infected cells. In this case, a faster virophage spread due to a lower death rate of these infected cells impairs the primary virus, which in turn increases the equilibrium level of the host amoeba. Thus, a lower virophage-induced death rate of cells increases the host population (Fig. 1). In contrast, when *f* >> 0, then a significant amount of primary virus replication still occurs in virophage-infected cells, and we see a one humped relationship (Fig. 1). For higher virophage-induced death rates of cells, *a*_ph_, a reduction in *a*_ph_ leads to larger host equilibrium levels as before. The inhibition of the primary virus, which benefits the host, is the dominant effect here. For lower levels of virophage-induced death of cells, however, the trend reverses and lower values of *a*_ph_ lead to lower host amoeba equilibrium levels. Now the higher yield of the primary virus, brought about by the reduced rate of virophage-induced cell death, is the dominant factor and negatively impacts the host population.

**Figure 2:**
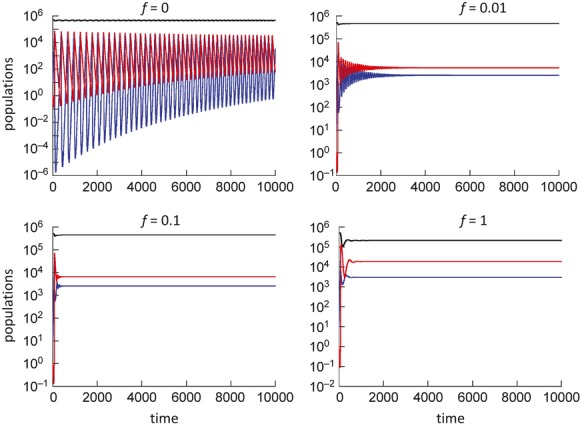
Dynamics predicted by model (1), depending on the virophage pathogenicity, *f*. The host population is shown in black, cells infected with the primary virus in blue, and cells infected with the virophage in red. The more the virophage inhibits the primary virus (lower *f*), the more unstable the dynamics become, leading to more extensive oscillations and longer damping times. Parameters were chosen as follows. *r* = 0.01; *β*_1_ = 2.5 × 10^−7^; *a*_1_ = 0.01; *β*_2_ = 2 × 10^−5^; *a*_ph_ = 0.05; *k* = 5 × 10^5^.

As discussed above, if the basic reproductive ratios of the primary virus and the virophage are greater than one, and if *r* > 0, then the equilibrium describing the persistence of the two viruses and the host is stable. The equilibrium is approached by damped oscillations, with the damping time and the extent of the oscillations depending on the model parameters. Previous work on hyperparasitism has shown that the introduction of the hyperparasite can both have a stabilizing and a destabilizing effect on the dynamics. We examined how the degree of virophage-mediated primary virus inhibition (i.e., the “virophage pathogenicity”) influences the approach to equilibrium (Fig. 2). The most pronounced oscillations and the longest damping times are observed for maximal virophage pathogenicity, that is, if the degree of primary virus inhibition is maximal such that *f* = 0 (Fig. 2). Reducing the degree of virophage pathogenicity (increasing *f*) greatly stabilizes the dynamics, leading to significantly shorter damping times (Fig. 2). Thus, higher degrees of virophage pathogenicity correlate with less stable dynamics.

**Figure 3:**
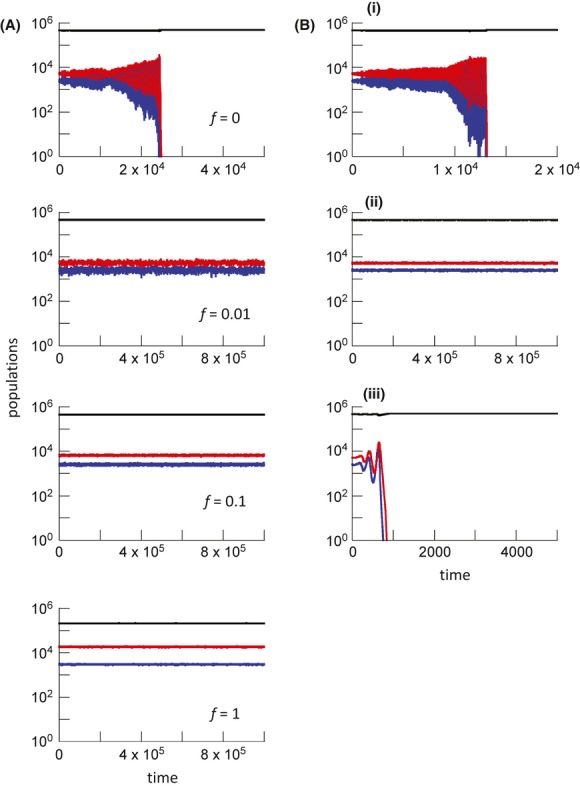
(A) Dynamics predicted by the stochastic, Gillespie simulation of ODE system (1), depending on the virophage pathogenicity, *f*. The host population is shown in black, cells infected with the primary virus in blue, and cells infected with the virophage in red. Figure 2 showed that dynamics become more unstable for lower *f*. Here, simulations were started at the equilibrium levels predicted by the ODEs (the nearest integer number) and typical outcomes were plotted. Starting around the equilibrium minimizes the chances of extinction due to oscillatory dynamics. For *f* = 0, the dynamics are the most unstable and the system crashes to extinction. Higher values of *f* stabilize the dynamics, resulting in long-term persistence. Parameters were chosen as follows: *r* = 0.01; *β*_1_ = 2.5 × 10^−7^; *a*_1_ = 0.01; *β*_2_ = 2 × 10^−5^; *a*_ph_ = 0.05; *k* = 5 × 10^5^. (B) Gillespie simulation of the ODE system (1a), which takes free virus populations into account explicitly. All simulations assume maximal virophage pathogenicity, *f* = 0. The turnover of the free virus populations is varied, while keeping their basic reproductive ratios identical. (i) Baseline scenario, where *η*_1_ = 10; *u*_1_ = 10; *η*_2_ = 10; *u*_2_ = 10. Extinction occurs relatively quickly, similar to model (1) which assumed free virus to be in a quasi steady state. (ii) The turnover of the primary virus was reduced such that the death rate of free viruses is on the same order of magnitude as that of infected cells, that is, *η*_1_ = 0.01; *u*_1_ = 0.01; *η*_2_ = 10; *u*_2_ = 10. More stable dynamics and long-term persistence are observed. (ii) The turnover of the virophage population is reduced such that the death rate of virophages is of the same order of magnitude as that of infected cells, that is, *η*_1_ = 10; *u*_1_ = 10; *η*_2_ = 0.01; *u*_2_ = 0.01. This destabilizes the dynamics, accelerating extinction.

If oscillatory dynamics occur, population extinction can be observed in a stochastic setting. This was shown by performing stochastic, Gillespie simulations of the ODEs (Fig. 3A). The details of this methodology are well documented (Gillespie 1977). The parameters and cases considered are equivalent to those in Figure 2. The stochastic simulations were started at the integer population levels that are closest to the equilibrium numbers predicted by the ODEs, as this minimizes the extent of oscillations. Nevertheless, we observe quick extinction of the primary virus and the virophage for *f* = 0, that is, for maximally pathogenic virophages (Fig. 3). Long-term persistence was observed for higher values of *f*.

The model examined so far assumes that the free virus population is in a quasi steady state and is not explicitly taken into account. This assumption is often made in the context of virus dynamics because the turnover of the virus population tends to be much faster than that of the target cell population. However, this assumption need not always be true. For example, sediments appear to be a long-term reservoir for infective viruses of the marine alga Heterosigma akashiwo (Lawrence et al. 2002). It is conceivable that such reservoirs also apply to mimiviruses. Hence, we consider a model that explicitly tracks the free virus populations. While the equilibria and their stability remain the same, the dynamics with which the persistence equilibrium is approached can be different, and this can have implications for the ability of the virophage–primary virus–host system to persist. The model is given by the following set of ODEs.


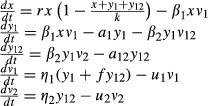
(1a)

The primary virus population is denoted by *v*_1_ and the virophage population by *v*_2_. Primary viruses are produced by cells infected with primary virus only with a rate *η*_1_, and by cells containing both the primary virus and the virophage with a reduced rate *fη*_1_. They decay with a rate *u*_1_. Virophages are produced from virophage-infected cells with a rate *η*_2_ and decay with a rate *u*_2_. The situation of maximum virophage pathogenicity, *f* = 0, will be examined in the context of the stochastic Gillespie algorithm. The same parameter combination as in Figure 3A will be considered, and the turnover of the primary virus will be varied (*η*_1_ and *u*_1_) while keeping the basic reproductive ratio of the primary virus constant. If the primary virus turnover is much larger than the turnover of infected cells (relatively large values of *η*_1_ and *u*_1_), the dynamics are similar compared with the quasi–steady state assumption, that is, pronounced oscillations are observed and extinction occurs relatively fast (Fig. 3B, i). However, if the turnover of free virus is lower and of the same order of magnitude as the turnover of infected cells, then more stable dynamics are observed that result in long-term persistence (Fig. 3B, ii). On the other hand, if the turnover of the virophage population is reduced such that the life span of the virophage is of the same order as that of infected cells, then population oscillations are amplified, promoting early extinction of the populations (Fig. 3B, iii).

## Evolution of Virophage Pathogenicity

Here, we examine the evolutionary dynamics of the virophage and concentrate in particular on the evolution of “virophage pathogenicity,” defined by the parameter *f*, describing the degree to which the primary virus can replicate when infected with the virophage. We introduce a second virophage strain into model (1), returning to the quasi–steady state assumption for the free virus populations. The model is now formulated as follows:


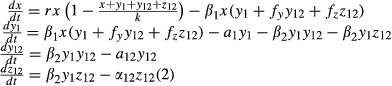
(2)

**Figure 4:**
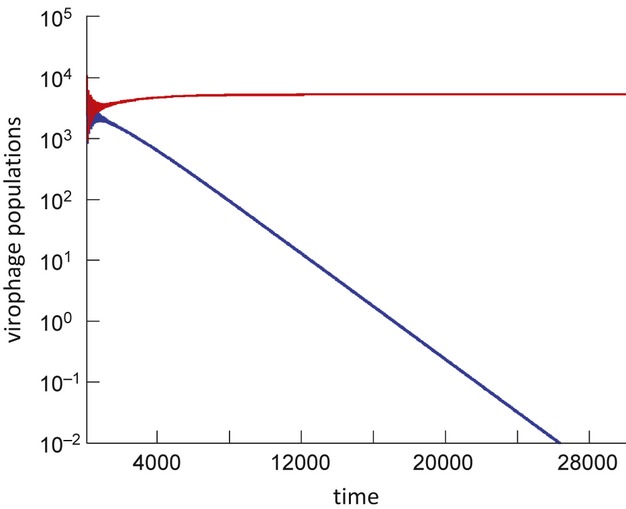
Virophage competition, according to model (2). The red line depicts the virophage population with a higher pathogenicity (lower *f*), whereas the blue line depicts the virophage population with a lower pathogenicity. The virophage with the higher pathogenicity (lower *f*) wins the competition. Parameters were chosen as follows: *r* = 0.01; *β*_1_ = 2.5 × 10^−7^; *a*_1_ = 0.01; *β*_2_ = 2 × 10^−5^; *a*_ph_ = 0.05; *f*_*y*_ = 0.05; *f*_*z*_ = 0; *k* = 5 × 10^5^.

Cells containing the primary virus can become infected by two virophage strains, and the respective virophage-infected cells are denoted by y_12_ and z_12_. The two strains only differ in their pathogenicity, which is denoted by *f*_*y*_ and *f*_*z*_. The death rate of these infected cells is thus given by *a*_12_ = *a*_ph_ + *f*_*y*_*a*_1_ and *α*_12_ = *α*_*ph*_ + *f*_*z*_*a*_1_ (note that the death rate of cells infected with the second strain is different and given by a greek letter). The basic reproductive ratio of virophage strain 1 is given by 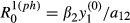 or 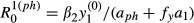. The expressions for strain 2 is 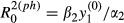 or 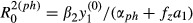. Because increased pathogenicity reduces the replication of the primary virus, it also increases the life span of the infected cell. This in turn leads to a higher total viral output of the virophage and thus to a higher basic reproductive ratio. In this model, the virophage strain with the higher basic reproductive ratio wins the competition, as demonstrated in Figure 4. Hence, the virophage population is expected to evolve to maximum pathogenicity, that is, to *f* = 0.

**Figure 5:**
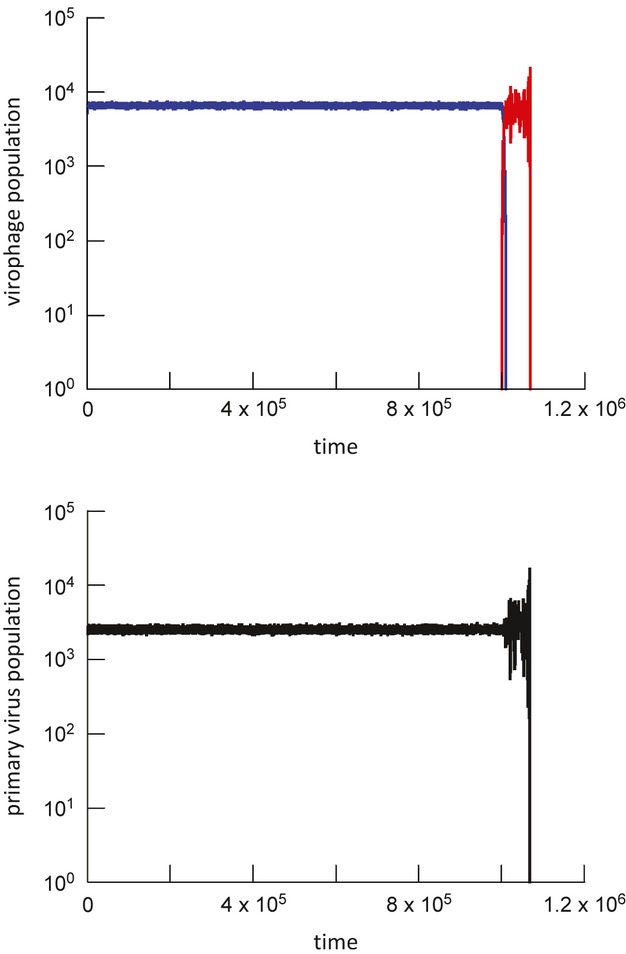
Evolution of the virophage to a higher degree of pathogenicity can lead to population extinction, according to Gillespie simulations of model (2). The simulation is started with the first virophage strain (blue) around equilibrium. The second virophage strain with increased pathogenicity (red) is subsequently introduced, invades, and excludes its competitor. Now the dynamics start to oscillate (due to the higher level of virophage pathogenicity), and the population crashes to extinction. Parameters were chosen as follows: *r* = 0.01; *β*_1_ = 2.5 × 10^−7^; *a*_1_ = 0.01; *β*_2_ = 2 × 10^−5^; *a*_ph_ = 0.1; *f*_*y*_ = 0.05; *f*_*z*_ = 0; *k* = 5 × 10^5^.

As shown in the previous section, an increase in virophage pathogenicity can lead to more extensive population oscillations and longer damping times, with *f* = 0 characterized by the most unstable dynamics. This can render populations prone to extinction, and these aspects were explored with stochastic Gillespie simulations of the ODEs. Figure 5 shows a scenario where a virophage strain with increased pathogenicity invades the population, and displaces the competing strain. The ensuing population oscillations quickly drive the virophage population extinct, and the primary virus can also be driven to extinction in this process. Thus, while selection favors a virophage strain with increased pathogenicity, the population can evolve to a state in which it is very prone to extinction.

**Figure 6:**
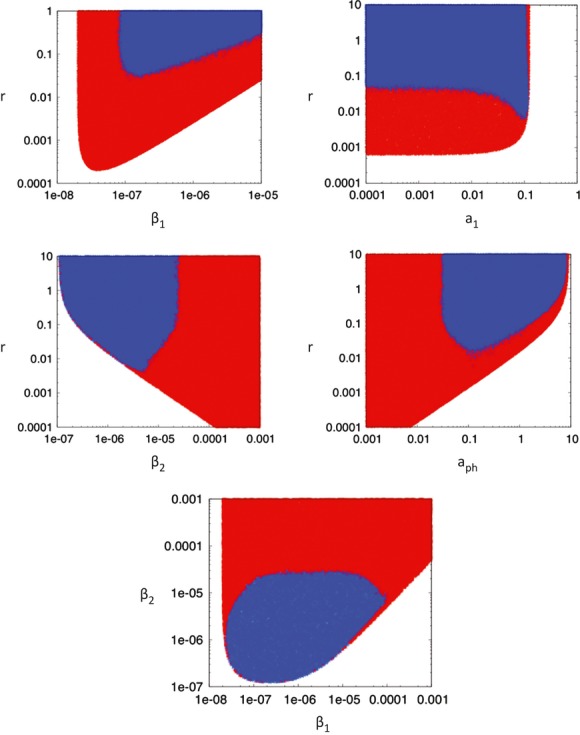
Extinction versus persistence of a virophage with maximal pathogenicity (*f* = 0) in dependence of model parameters. The graphs are based on Gillespie simulations of model (2). Simulations were started at the equilibrium (nearest integer number) according to ODE model (1). The simulations were run until a time threshold of 50,000 time units, and it was recorded whether the populations were extinct (red) or persisted (blue). The parameters indicated in the plots were randomly varied 100,000 times. Note that the borders between extinction and persistence can be fuzzy due to randomness in the outcomes. The exact picture depends on the time threshold when the simulation is stopped. Obviously, any stochastic simulation will end in extinction if it is run for long enough, irrespective of the parameters. However, in the blue parameter region, persistence lasts for a significantly longer time than in the red region. Base parameters were chosen as follows: *r* = 0.01; *β*_1_ = 2.5 × 10^−7^; *a*_1_ = 0.01; *β*_2_ = 2 × 10^−5^; *a*_ph_ = 0.05; *k* = 5 × 10^5^.

Whether extinction occurs for maximally pathogenic virophages (*f* = 0) depends on the model parameters and this is explored systematically in Figure 6. Obviously, whether extinction occurs or not can depend on the initial conditions, but is least likely if the simulation is started around the equilibrium values. Hence, starting from the equilibrium (at the nearest integer number, as the simulation is stochastic), the simulation was run for a defined period of time and it was recorded whether virophage extinction occurred during this time frame. This was done for different parameter combinations and the outcome is color coded in Figure 6. Persistence requires that the equilibrium population levels are sufficiently high such that the oscillatory dynamics do not lead to extinction. In this respect, the equilibrium number of primary virus–infected cells, *y*_1_, is of particular importance. If the virophage drives this population extinct, then it depletes its own targets for replication. High population levels of primary virus–infected cells, and thus persistence, are promoted by slow spread of the virophage, that is, by a slow virophage replication rate, *β*_2_, and a fast virophage-induced cell death, *a*_ph_ (Fig. 6). In addition, persistence is promoted by a fast growth rate of the host amoeba population, *r* (Fig. 6). The replication rate of the primary virus, *β*_1_, and the rate of cell death induced by the primary virus, *a*_1_, only have relatively small effects on the outcome (Fig. 6). Because the virophage will likely evolve toward faster replication kinetics, this suggests that evolutionary trajectories will bring the system into a parameter regime that renders the populations prone to extinction.

## Evolution of the Primary Virus

Here, the evolutionary dynamics of the primary virus are investigated, concentrating on the viral replication rate, *β*_1_, and the rate of virus-induced cell killing, *a*_1_. A model with two primary virus strains is considered that compete for the same host population. Cells infected with the second strain of the virus are denoted by equivalent capital letters, that is, cells infected with the second strain of the primary virus only are denoted by *Y*_1_, and cells that also contain the virophage by *Y*_12_.


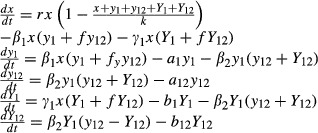
(3)

The infection rate of the second strain primary virus is given by *γ*_1_, and the death rate of cells infected with the second strain primary virus is given by *b*_1_ in the absence of the virophage and *b*_12_ in the presence of the virophage (where *b*_12_ = *a*_ph_ + *fb*_1_). The basic reproductive ratio of the first strain is the same as before, that is, 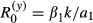, and that of the second strain is given by 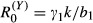.

In the absence of the virophage, the primary virus strain with the larger basic reproductive ratio wins the competition, and thus evolution will maximize the basic reproductive ratio (subject to constraints that are not included in this model).

**Figure 7:**
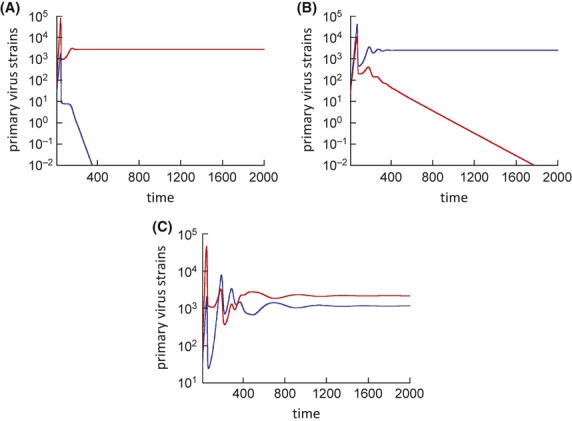
Competition between two primary virus strains in the presence of the virophage, according to model (3). The population of the second strain shown in red has a lower basic reproductive ratio, *R*_0_, than the first strain shown in blue. As can be seen from the graphs, the strain with the lower basic reproductive ratio can win the competition (A), lose the competition (B), or coexistence can be observed (C), depending on the parameters. Parameters were chosen as follows: *r* = 0.01; *β*_1_ = 2.5 × 10^−7^; *a*_1_ = 0.01; *β*_2_ = 2 × 10^−5^; *a*_ph_ = 0.05; *f* = 0.1; *k* = 5 × 10^5^; (A) *γ*_1_ = 2 × *β*_1_; *b*_1_ = 5 × *a*_1_; (B) *γ*_1_ = 2 × *β*_1_; *b*_1_ = 15 × *a*_1_; (C) *γ*_1_ = 10^−6^; *b*_1_ = 0.3.

**Figure 8:**
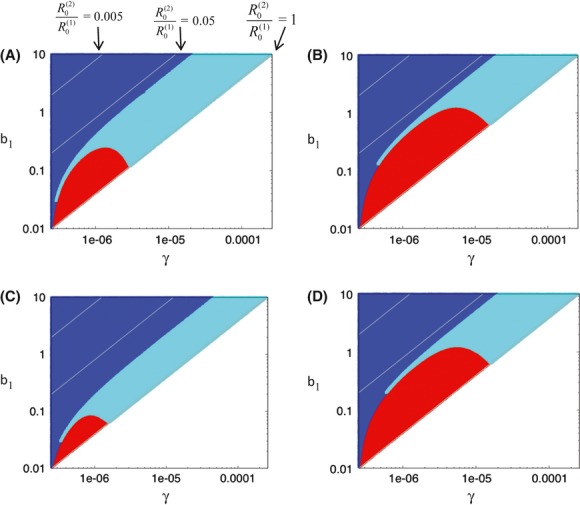
Outcome of competition between two primary virus strains in the presence of the virophage, depending on the parameter values, according to model (3). Strain 2 is assumed to have a lower basic reproductive ratio, *R*_0_, than strain 1. Strain 2 with the lower *R*_0_ wins in the red parameter region. Strain 1 wins in the blue parameter region. Coexistence is observed in the cyan parameter region. The gray lines indicate the ratio of *R*_0_ for strain 2 over that of strain 1. The lower this ratio, the lower the relative *R*_0_ of strain 2. The gray lines show that the ratio of 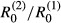 per se does not determine the outcome of competition (on the lines, the ratio is identical). Different outcomes can be observed for the same ratio 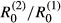. The different graphs show the parameter exploration for different parameter values. Panel (A) is the base scenario. Panel (B) assumes a stronger virophage due to a faster virophage replication rate. Because the virophage is stronger, the parameter region in which the primary virus with the lower *R*_0_ wins is larger. Panel (D) also shows a stronger virophage, this time indirectly due to a faster replication rate of the host population, demonstrating a similar effect. Panel (C) is done for a relatively low virophage pathogenicity, that is, a high value of *f*. This reduces the life span of infected cells because of less inhibition of primary virus replication, thus lowering the virophage burst size. Consequently, the parameter region in which strain 2 with the lower *R*_0_ wins is reduced. Base parameters were chosen as follows: (A) *r* = 0.01; *β*_1_ = 2.5 × 10^−7^; *a*_1_ = 0.01; *β*_2_ = 2 × 10^−5^; *a*_ph_ = 0.1; *f* = 0.1; *k* = 5 × 10^5^. (B) Same as (A) except *β*_2_ = 2 × 10^−4^. (C) Same as in (A) except *f* = 1. (D) Same as in (A) except *r* = 0.1.

The situation is more complex in the presence of the virophage. If a strain is characterized only by a higher replication rate (*β*_1_ or *γ*_1_) it always wins the competition. The most obvious reason is that a faster replication rate increases the basic replicative fitness of the virus. In addition, however, a faster replication rate of the primary virus indirectly conveys a benefit by weakening the virophage. As shown in equilibrium expression 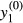, a faster replication rate of the primary virus reduces its equilibrium level in the absence of the virophage, and thus reduces the basic reproductive ratio of the virophage. In fact, weakening the virophage can be more important than increasing the basic replication kinetics of the primary virus. This is illustrated as follows. Assume that the second primary virus strain replicates faster (*γ*_1_ > *β*_1_) and that it is also characterized by a higher death rate of infected cells (*b*_1_ > *a*_1_). Further assume that the increase in the death rate of infected cells is greater than the increase in the viral replication rate. In this case, the basic reproductive ratio of the second primary virus strain, 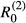, is lower than that of the first strain, 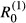; this also lowers the spread rate of the virophage. Under these assumptions, three outcomes are possible (Fig. 7). As expected, the strain with the larger *R*_0_ can win the competition. Interestingly, the strain with the smaller *R*_0_ can also win and exclude its competitor. Alternatively, coexistence of the two strains can be observed. The dependence of the outcomes on parameters is explored in Figure 8A. Coexistence occurs only if *γ*_1_ >> *β*_1_. If 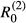 lies below a threshold, the second strain fails to invade and goes extinct. If 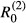 is higher but still below the value of 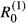, then the second strain can invade and exclude the first strain. The more effective the virophage is, the larger the parameter space in which the primary virus with the lower *R*_0_ excludes the strain with the higher *R*_0_. This is shown in Figure 8B–D by exploring the parameter space for different scenarios that vary in the effectiveness of the virophage. Therefore, if the virophage has a significant negative impact on the primary virus population, selection can favor primary viruses with a reduced *R*_0_ because it lessens the impact of the virophage. In other words, the presence of the virophage can lead to evolution toward reduced replicative fitness of the primary virus.

## Discussion and Conclusion

This study used mathematical models to study the dynamics between a host population, its primary virus, and a virophage infecting the primary virus. In particular, the model was built with the *Acanthamoeba*–mimivirus–sputnik system in mind, although population dynamic measurements or parameter measurements that would allow a closer application are currently not available. Ecological studies point to the importance of virophages in regulating primary viruses and thus impacting protist populations (Yau et al. 2011). In the context of one specific study, a Lotka–Volterra-type mathematical model was used to underline this point (Yau et al. 2011) in the context of Antarctic lake protists. However, a more general exploration of the dynamics has not been provided. This was done here, with an emphasis on the evolutionary dynamics. Not surprisingly, some of the basic properties of the model are very similar to those observed in models of hyperparasitism (Beddington and Hammond 1977; May and Hassell 1981; Hochberg et al. 1990; Holt and Hochberg 1998). For example, by regulating the primary virus population, the virophage can have a positive effect on the host amoeba population. However, as in the current model the virophage-infected cells can still allow transmission of the primary virus to host cells, a reduction in the virophage-induced death rate of cells, and thus a faster spread of the virophage, can also negatively impact the amoeba host population. A lower virophage-induced death rate of cells not only allows release of more virophages but also of more primary virus. In general, while the models considered here are closely related to previously studied hyperparasitism models, they do differ in aspects that specifically apply to the infection of primary viruses by virophages. Thus, as mentioned above, the presence of both viruses in the same host cell can lead to interactions that simultaneously influence the total number of each virus released from the host cell during its life span. In addition, the virophage can only parasitize the primary virus in the intracellular stage during replication and not at the free virus stage, a distinction that does not necessarily apply to general models of hyperparasitism.

Beyond the basic dynamics, some interesting evolutionary insights emerged. While the virophage is expected to evolve toward higher levels of primary virus inhibition, this can lead to more oscillatory dynamics which can result in extinction of the virophage and also the primary virus. For pathogenic virophages, persistence is only possible for relatively slow virophage replication kinetics, which is again not favored by evolution. This theoretical result brings up the question whether the presence of a virophage in food chains is transient and eventually destined to go extinct as a result of virophage evolution itself. Data, however, argue against this notion. Sputnik appears to have a long evolutionary history with Mimi-virus (Claverie and Abergel 2009; Sun et al. 2010), which is thought to be as ancient as Eucarya (Iyer et al. 2006). Also, virophages have persisted in other giant viruses of the mimivirus lineage (Fischer and Suttle 2011; Yau et al. 2011). This suggests a stable evolutionary association between virophage, virus, and host. In the light of the theory presented here, this could be achieved by a variety of mechanisms. The extended model (1a), which explicitly took into account free virus populations, suggests that different viral turnover strategies can have an important influence on the stability of the system, which in turn influences how prone the system is to extinction. If the primary virus achieves reproductive success by having a long-lived infectious-free virus stage (e.g., through reservoirs in sediments (Lawrence et al. 2002)) rather than investing in a high rate of reproduction in cells, the dynamics become significantly more stable which could ensure evolutionary persistence in the presence of the virophage. Another important aspect that was not considered in this study is spatially structured populations. The model presented here, given by ODEs, assumes perfect mixing of all populations. While this is an assumption often made in the context of virus dynamics (Nowak and May 2000), it might apply best to in vitro situations and perhaps less so to natural populations, where spatial interactions often play important roles (Briggs and Hoopes 2004). This argument is similar to that in the context of the more straightforward predator–prey dynamics, which can be unstable and prone to extinction in a perfectly mixed setting, but more stable and persisting in the long term in the presence of spatial structure. The interactions explored here are based on Lotka–Volterra-type predation dynamics, where the stabilizing effect of space has been extensively demonstrated (Briggs and Hoopes 2004). Another aspect of the model presented here that might be unrealistic is the assumption that the virophage can indeed evolve toward sufficiently high levels of pathogenesis (low values of *f*) such that the dynamics become unstable enough to cause extinction in stochastic settings. It is possible that there are constraints that limit the degree to which the virophage can inhibit primary virus replication, thus preventing the virophage to evolve to a state that would leave the system prone to extinction. It is currently not known whether such constraints exist, but it is feasible from a biological point of view, for example, if there is a trade-off between the ability of the virophage to inhibit the primary virus and its ability to reproduce in the viral factories maintained by the primary virus.

With respect to the evolution of the primary virus, the model suggests that the presence of virophages can fundamentally alter the evolutionary course, selecting for primary viruses with a reduced basic reproductive ratio. This in turn could allow the ecosystem to evolve to a state that is beneficial for the host amoeba population. Thus, the virophage may not only benefit the host amoebae directly by attacking the primary virus, but it may also do so indirectly by influencing the course of primary virus evolution.

While our model was constructed specifically with virophages in mind, it could potentially also apply to satellite viruses in general. There is a debate in the literature whether virophages represent a new class of viruses or whether they are part of the larger group of satellite viruses that require the help of another virus for replication (Herrero-Uribe 2011; Krupovic and Cvirkaite-Krupovic 2011; Desnues and Raoult 2012; Fischer 2012). It has been argued that some satellite viruses can also negatively impact the helper virus (Krupovic and Cvirkaite-Krupovic 2011). However, the model discussed here examines the role of virophage “pathogenicity” where the virophage can have a substantial impact on the fitness of the primary virus. Unless this assumption applies to satellite viruses, the applicability of the model presented here is limited.

It is important to also point out uncertainties and limitations of this analysis. Relatively simple equations were used to study the dynamics and evolution of the virophage–primary virus–host system. As with all models, the conclusions can depend on the exact assumptions and model formulation. In the context of natural populations, the most striking simplifying assumption is that all populations mix perfectly, which is inherent in the ODE formulation that is also used in a large portion of the literature on virus dynamics. This is probably an accurate description of in vitro experiments, but might be less realistic for natural populations where spatial restrictions can play important roles. The effect of spatial structure on the evolutionary dynamics should be investigated in future work. However, describing a simplified scenario that is more likely to apply to in vitro experiments is still a very important step in the investigation because it is possible to test the model by experiments and because this also forms the basis for more complex models that take into account spatial structure (e.g., through metapopulation models). Even within the perfect mixing assumption, uncertainties remain in the model because the same process can be formulated in different ways. For example, consider the infection term, which is generally given by *rate constant x number of target cells x number of infected cells*. While this is the most widely used term in the context of virus dynamics and also epidemiological models (Anderson and May 1991; Nowak and May 2000), different mathematical descriptions can be used, for example, terms that saturate in the number of target cells and/or infected cells (McCallum et al. 2001; Wodarz and Komarova 2009). This could change certain model properties. Therefore, it is important to perform in vitro experiments to test whether the model used here can successfully describe experimental data that document the time evolution of an appropriate system, such as the amoeba–mimivirus–sputnik system. If the model presented here is able to successfully describe such data, it is at least consistent with data and can be used for further developments, introducing more complex biological assumptions. Often, however, the value of a model lies in the disagreement with experimental data. In this case, it is possible to reject particular assumptions with certainty and to narrow the search for the correct description.

The basic model validity can be tested by very simple in vitro experiments where a host *Acanthamoeba* population is infected with both the mimivirus and the virophage at different initial concentrations. Nonlinear least squares fitting procedures can be used to see how well the system can describe the data, and to estimate parameters in the context of one set of initial conditions. By changing the initial concentrations of cells and viruses, the parameterized model should then successfully predict subsequent experiments. Once the model has been validated/revised, more complex experiments can be performed to address specific model predictions discussed in this study. For example, conditions can be altered such that growth and death parameters are changed, or the cells and viruses could be manipulated to have the same effect. The influence of these parameter manipulations on the stability of the dynamics should be observed. This would provide an important piece of information to advance our understanding of the evolutionary dynamics discussed here. This in turn will be important for a better understanding of the microbial ecology and the evolutionary dynamics of aquatic and marine systems.
